# Fine control of chlorophyll-carotenoid interactions defines the functionality of light-harvesting proteins in plants

**DOI:** 10.1038/s41598-017-13720-6

**Published:** 2017-10-24

**Authors:** Vytautas Balevičius, Kieran F. Fox, William P. Bricker, Sandro Jurinovich, Ingrid G. Prandi, Benedetta Mennucci, Christopher D. P. Duffy

**Affiliations:** 1 School of Biological and Chemical Sciences, Queen Mary University of London, London, E1 4NS UK; 2 Department of Biological Engineering, Massachusetts Institute of Technology, Cambridge, MA 02139 USA; 3 Dipartimento di Chimica e Chimica Industriale, University of Pisa, Via G. Moruzzi 13, Pisa, 56124 Italy; 4 Department of Chemistry, Federal University of Lavras, 37200-000 Lavras, Brazil; 5 Laboratory of Molecular Modeling Applied to the Chemical and Biological Defense, Military Institute of Engineering, Praça Gen, Tibúrcio, 80 Rio de Janeiro Brazil

## Abstract

Photosynthetic antenna proteins can be thought of as “programmed solvents”, which bind pigments at specific mutual orientations, thus tuning the overall energetic landscape and ensuring highly efficient light-harvesting. While positioning of chlorophyll cofactors is well understood and rationalized by the principle of an “energy funnel”, the carotenoids still pose many open questions. Particularly, their short excited state lifetime (<25 ps) renders them potential energy sinks able to compete with the reaction centers and drastically undermine light-harvesting efficiency. Exploration of the orientational phase-space revealed that the placement of central carotenoids minimizes their interaction with the nearest chlorophylls in the plant antenna complexes LHCII, CP26, CP29 and LHCI. At the same time we show that this interaction is highly sensitive to structural perturbations, which has a profound effect on the overall lifetime of the complex. This links the protein dynamics to the light-harvesting regulation in plants by the carotenoids.

## Introduction

Photosystem I (PSI) and Photosystem II (PSII) are large, integral membrane protein super-complexes in plants and green algae^1,2^. They are the key components of the light reactions of photosynthesis. While PSII performs water oxidation to build a transmembrane proton gradient and induce electron transfer^3^, PSI primarily produces the universal redox carrier NADPH^4^. From the functional perspective, the photosystem super-complexes are divided into the core sites of actual photochemical reactions, called reaction centers (RCs), and accessory light-harvesting complexes (LHCs). Even though the RC sub-units capture light themselves, the peripheral LHC antenna proteins of the Lhcb (in PSII) and Lhca (in PSI) families are necessary to increase the absorption cross-section and ensure optimal performance^5^. The antenna complexes transfer excitation energy with remarkable efficiency, enabling near unity quantum yield of PSI/II (one photochemical reaction per one photon captured)^6^. This is due to the fine tuning of the relative positions, orientations and excitation energies of chlorophyll (Chl) cofactors coordinated by the residues, which is the reason why LHC proteins are sometimes referred to as “programmed solvent”^2^. The current precision of pigment placement resolved from the crystal structures^7–10^ allows for highly detailed models, describing both the initial steps of exciton transfer^11,12^ and the subsequent charge separation in the RCs^13^. However, such models largely account only for the Chls, while the second major building block of the pigment arrays, the carotenoids (Cars), are usually disregarded.

Cars are typically included in photodynamic models of bacterial systems only, where they are significant light-harvesters^14^, while their light-harvesting role in plants is minor compared to the photoprotective function^15,16^. The latter is performed primarily by quenching the Chl triplet states^17^, which would otherwise sensitize molecular oxygen to form harmful singlet oxygen species^18^. Alternatively, Cars may directly act as singlet oxygen scavengers^19^. However, an accumulating body of knowledge points to even deeper involvement of Cars in photoprotection, already suppressing the formation of Chl triplet states: a part of a process termed non-photochemical quenching (NPQ)^20^. Even though several molecular NPQ mechanisms are proposed^21^, Cars are especially appealing agent-candidates because of their extremely short-lived (10–25 ps) lowest singlet excited state S_1_^22^, which was demonstrated to yield significant quenching in artificial caroteno-phthalocyanine dyads^23^. However, the optically dark nature of S_1_^24^ makes it nearly impossible to be observed directly and poses considerable challenge in describing its properties from first principles^25^. These two features largely prevented the full inclusion of Cars in the photodynamic models in plants, an issue which is only currently being addressed^26,27^.

The fact that the lifetime of the S_1_ state is comparable to the typical time-scales of the energy transfer between the protein sub-units (or even shorter)^11^ raises a conceptual question: How can such pigments be incorporated into a light-harvesting system without hindering its function by wasteful dissipation of the captured energy? Or alternatively, how can such a dissipative channel play a specific integral role in regulating light-harvesting in a fluctuating light environment? In this study we analyze the nearest Chl–Car pairs in the plant antenna complexes LHCII/LHCI, CP26 and CP29 with particular focus on the mutual orientation. We emphasize the observation of repetitive conformation pattern within these systems. The study of the coupling strength between the lowest singlet excited states of the two pigments revealed that, within the phase-space of possible mutual orientations, the configuration of minimal coupling is assumed. Furthermore, we show that within such a configuration, Chl-to-Car excitation transfer rate is highly sensitive to the mutual orientation, which can be driven from excitation-preserving to quenching configurations within physiologically reasonable boundaries. This not only supports the idea of Cars acting as one of the agents regulating energy density in the photosystems under high-light conditions, but also presents the most feasible molecular switching pathway.

## Results

### Clearly-expressed preferred mutual orientation of closest co-facial Chl–Car pairs

**Figure 1:**
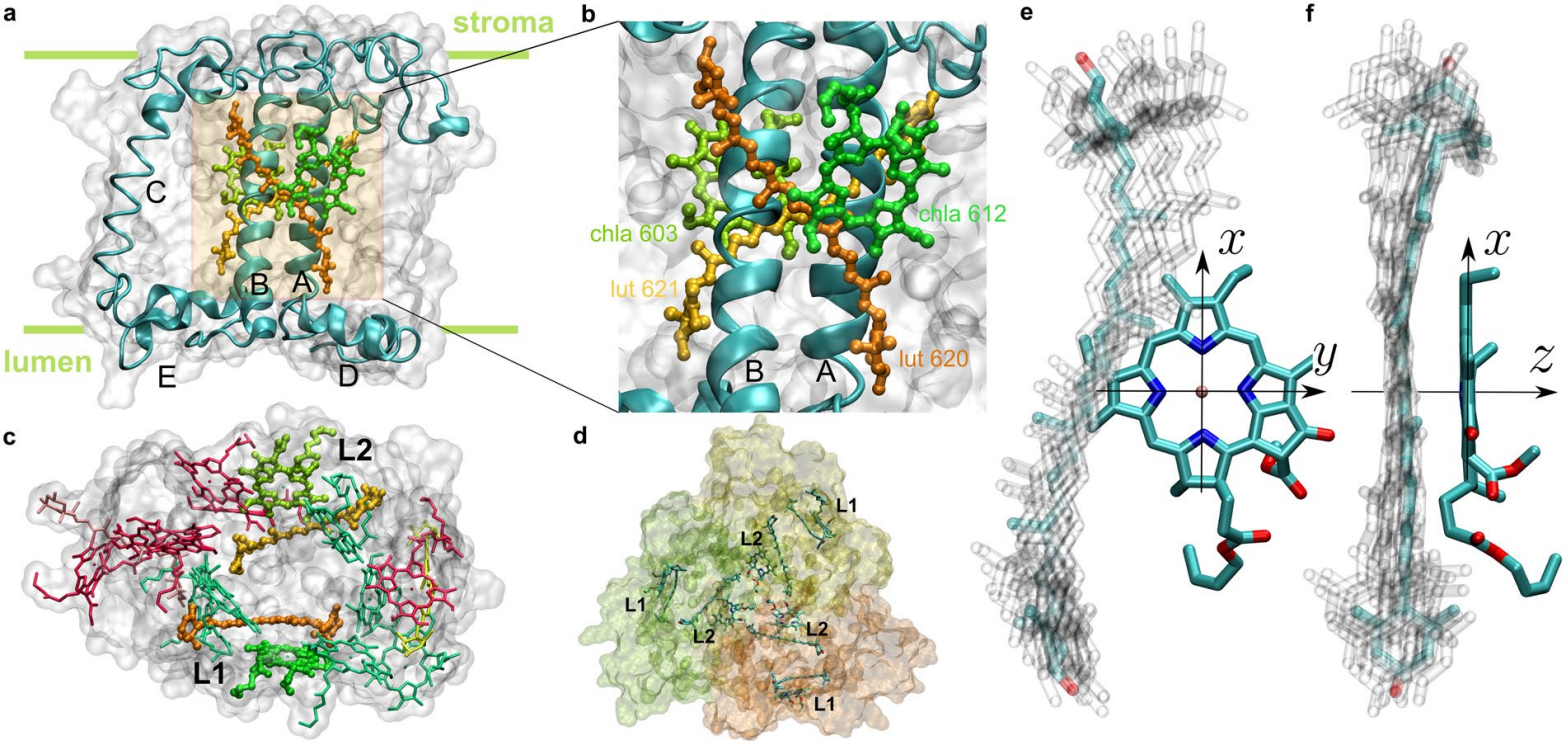
Mutual orientation of Cars and their closest co-facial Chl partners in the L1/L2 sites. (**a**–**c**) Positioning of Lhcb1 within the membrane (PDB: 3JCU)^9^. The views are along the membrane plane (**a**,**b**) and from the stromal side (**c**). A close-up view of the L1/L2 sites in b shows the pigment pairs and the naming nomenclature according to Liu *et al*.^7^. Transmembrane helices A, B, C and amphipathic helices D and E are shown in light-blue. The remaining pigment composition of Lhcb1 is shown in **c**: 8 Chl *a*’s are shown in green, 6 Chl *b*’s are in red, Vio and neoxanthin are shown in pink and yellow, accordingly (molecules and proteins plotted with VMD^31^). (**d**) The full trimer of the LHCII antenna (lumenal view). Monomers are emphasized by colors. (**e**,**f)** Superposition of all the L1 and L2 pairs from Lhcb1-5 and Lhca1-4 protein units. The front view (**e**) and the side view (**f**) also show the coordinate system associated with the Chl. Cars are shown as ghost atoms to emphasize the distribution rather than the actual positions, except for Lut 620 from L1 site of Lhcb1 which is highlighted for comparison. Only Chl *a*612 from the L1 site of Lhcb1 represents all the Chls for clarity because the other Chls from the pairs largely differ only by the conformation of their phytol tails, which do not contribute to the transition density (Supplementary Fig. 1a).

We inspected Car placement within the major and minor antenna proteins of the PSII supercomplex (PDB: 3JCU)^9^ and the antenna proteins of the PSI supercomplex (PDB: 4XK8)^8^. We focused on the Lhcb and Lhca sub-units that comprise LHCII (Lhcb1 (shown in Fig. 1a–c), Lhcb2 and Lhcb3 forming a trimer, Fig. 1d), CP29 (Lhcb4), CP26 (Lhcb5) and LHCI (Lhca1–4). Each of these homologous sub-units host several Car binding sites. We identify the Chls that are closest to the middle section of the Cars (C_16_=C_37_ bond), because that is the center of their transition density (which governs their coupling capability, see Supplementary Information). Additionally, we look for the Chl that has its chlorin ring maximally parallel to the conjugation plane of the Car, because that is a condition for maximal interaction due to the overlap of transition densities^28^. These conditions are best met by two Cars, each bound in an elongated groove on the two sides of the central transmembrane helices A and B of the Lhca/Lhcb apoproteins (Fig. 1a–c). The Car within the groove ending at the lumenal side helix D, called the L1 site (nomenclature according to Kühlbrandt *et al*.^29^), is assigned as lutein (Lut). The second site, L2, ending at the second short helix E, is occupied either by Lut (in Lhcb1–3 and 5) or by violaxanthin (Vio; in Lhcb4, Lhca1–4). The distance from Chl (central Mg atom) to Car (central bond) is between 5.7–6.7 Å in all Lhcas/Lhcbs.

Additionally, there are sites N1 (binds neoxanthin in Lhcbs1-5 or all-trans β-carotene in all Lhcas and, as recently suggested, Lhcb6^10^) and V1 (only in Lhcb1-3, possess either Vio, astaxanthin or zeaxanthin). However, since we are particularly interested in the possible role of Cars as singlet energy acceptors, we disregard these two sites. The N1 Cars are not taken into account since they interact almost exclusively with Chl *b*’s^30^ (which rapidly transfer energy to the Chl *a* pool), while the V1 site is disregarded because the surrounding Chls are only close to the Car end-groups, which barely contribute to the transition density of the S_1_ state (Supplementary Fig. 1b,c). Cars within V1 site were also shown to have their S_1_ state negligibly coupled to the nearest Chls because of unfavorable edge-on orientations and larger distances (>9 Å)^26^.

We have superimposed all L1 and L2 Chl–Car dimers onto one another, so as to achieve maximal coincidence of the four nitrogen atoms within the Chls (Fig. 1e,f). Interestingly, the orientations are strictly preserved among both the Lhcb/Lhca apoproteins and the two sites. Specifically, the Cars seem to be located at one preferred side of their Chl partner. In order to rationalize such preferred Chl and Car binding orientation, we investigated the dependence of the energy-transfer inducing electronic coupling on the mutual orientation of the two pigments. The coupling, also termed resonance interaction, between the lowest singlet states Chl Q_y_ and Car S_1_, ${J}_{{Q}_{y}{S}_{1}}$, was evaluated using the transition density cube (TDC) method^32^ (for precise definitions see Methods and Supporting Information). It was calculated under the rotation of Chl around the axis perpendicular to the chlorin ring and originating at the Mg atom (*z* axis, Fig. 1f). Since all the Cars and Chls assume slightly different molecular conformations enforced by the binding pockets we used planar (vacuum-optimized) structures superimposed onto the prototype Lut 620–Chl *a*612 and Lut 621–Chl *a*603 pairs from LHCII (nomenclature according to Liu *et al*.^7^).

**Figure 2:**
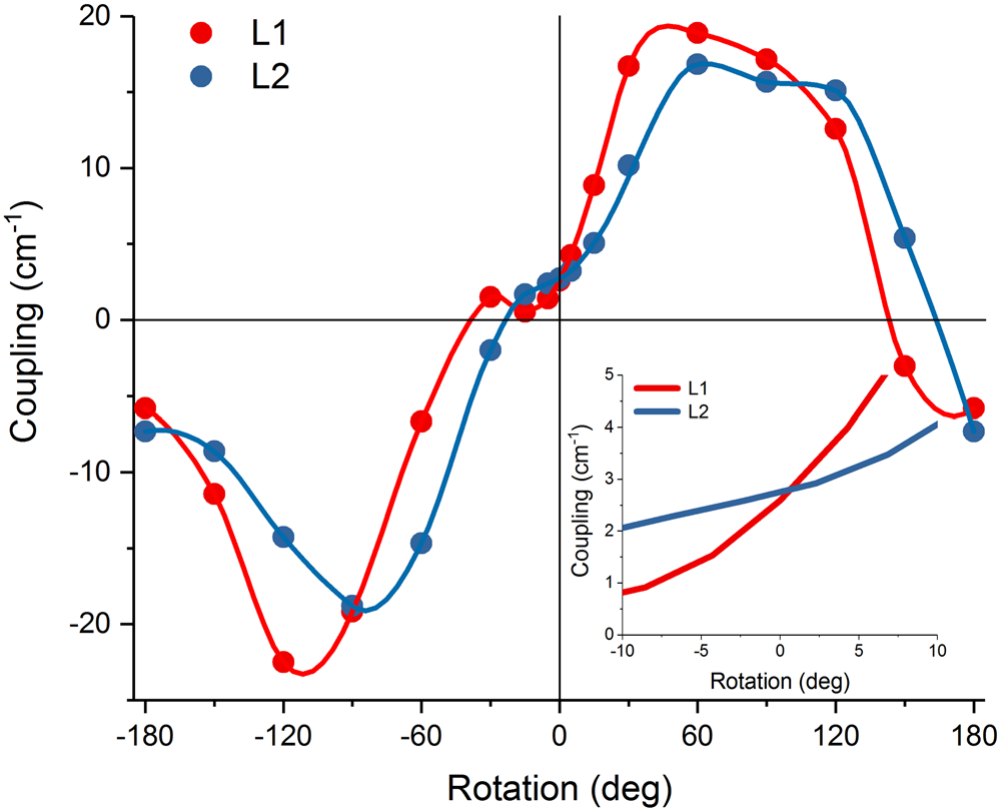
Chl–Lut resonance interaction ${{\boldsymbol{J}}}_{{{\boldsymbol{Q}}}_{{\boldsymbol{y}}}{{\boldsymbol{S}}}_{1}}$ as a function of the Chl rotation around the *z* axis. Results for L1 and L2 sites of a single Lhcb1 monomer (red and blue, accordingly) are qualitatively identical, except for a shallow local minimum for L1 site at −15°. Dots correspond to the calculated values, lines correspond to the spline interpolated values. The inset shows the dependence in the immediate vicinity of the original orientation at which the couplings are 2.6 cm^−1^ and 2.7 cm^−1^ for L1 and L2 sites, accordingly.

The resulting coupling dependence is shown in Fig. 2. The origin corresponds to the configuration of the planar molecules superimposed on the originals from the crystal structure. Results for both L1 and L2 pairs are shown to represent the effect of slight differences in the placement and initial orientation. The couplings are an order of magnitude smaller than their typical Chl–Chl counterparts (20–120 cm^−1^)^33^, which reflects the “dark” nature of the S_1_ state. The positive and negative peaks are reminiscent of the dipole–dipole interaction (sign is arbitrary). Most importantly, we notice that the actual orientation from the crystal structure corresponds to the minimal coupling (2.7 cm^−1^) between the two pigments. Furthermore, at this orientation the dependence demonstrates a shallow plateau as opposed to the steep dependence at the other *J* = 0 orientation.

### Flexibility of Chl–Car pairs in fluctuating environment

Having revealed the regularity of the Chl–Car orientational motif, a natural question arises: is such a configuration preserved *in vivo*, and if so, does it have physiological significance? Furthermore, it is important to know how stable and rigid such a configuration is. In order to study the extent of the configuration-space available to Cars with respect to the Chls we performed a Molecular Dynamics (MD) simulation of an LHCII trimer (Fig. 1d) within a lipid membrane. A 1 μs trajectory was generated and 1000 snapshots taken at every nanosecond are considered. Planar, vacuum-optimized Chl *a* and Lut were superimposed onto the extracted L1/L2 pairs (result for a random snapshot shown in Supplementary Fig. 3). This was done in order to have well quantum-chemically optimized molecules for coupling calculation. For details of the simulation and subsequent coupling calculations see the Methods and Supplementary Information.

**Figure 3:**
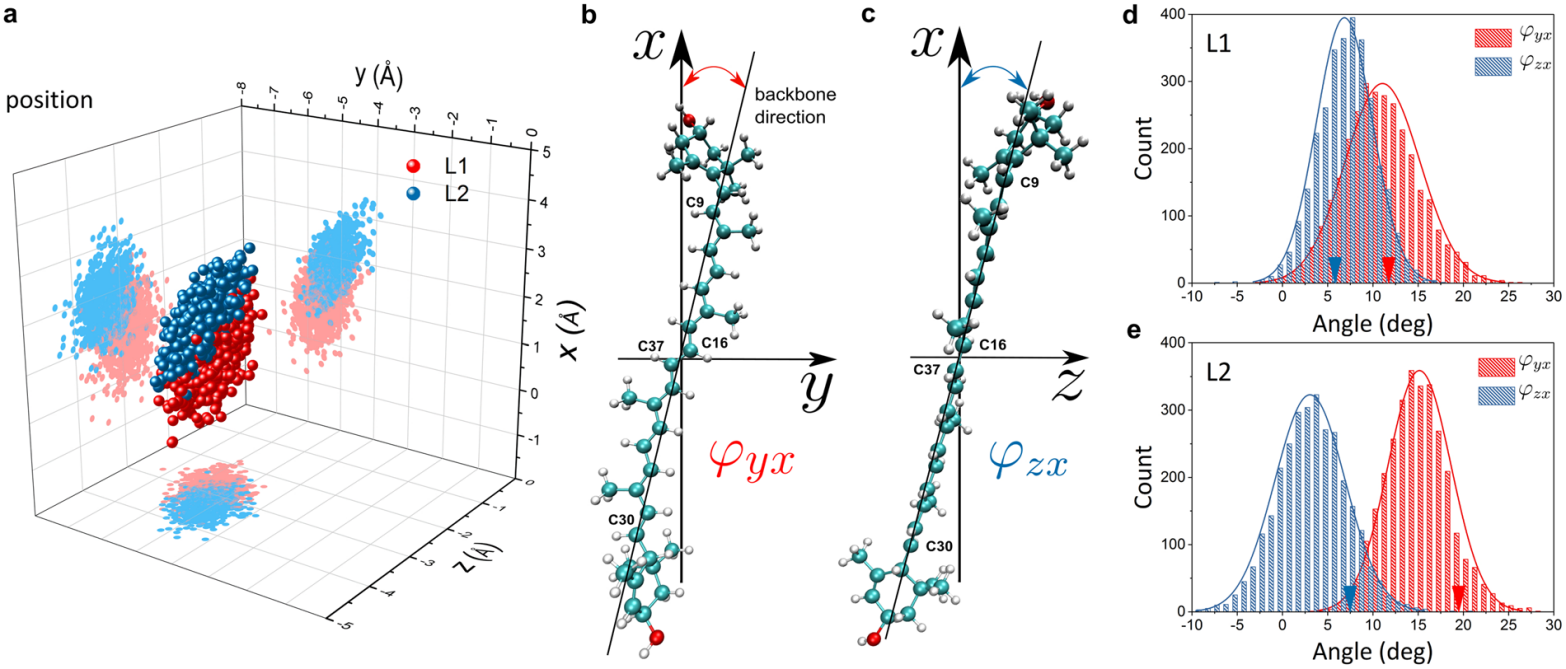
L1 and L2 pairs’ statistics from Molecular Dynamics simulation. (**a**) Distribution of the position of the central C_16_ = C_37_ bond of the two Luts within one monomer showing the distinction between L1 (red) and L2 (blue) sites. All coordinates and directions in this work are represented in the Chl *a* reference frame given in Fig. 1e,f. (**b**,**c**) Definition of the characteristic angles ${\phi }_{yx}$ and ${\phi }_{zx}$ that the Lut backbone (C_9_–C_30_ axis) projections form with the *x* coordinate axis: ${\phi }_{yx}$ angle corresponds to the projection of the backbone axis in the *xy* plane (**b**), likewise, ${\phi }_{zx}$ angle corresponds to the projection of the backbone axis in the *xz* plane (**c**). (**d**,**e**) The distribution of the ${\phi }_{yx}\,\,$and ${\phi }_{zx}$ angles for L1 (**d**) and L2 sites (**e**) within the full trimer. Distributions of ${\phi }_{yx}$ and ${\phi }_{zx}$ are shown in red and blue, accordingly. The arrows indicate the corresponding average values directly from the crystal structure PDB: 1RWT^7^ (averaged over all three trimers captured in the structure). The envelopes show the normal distribution fit.

The MD trajectory reveals that both Luts are rigidly fixed position-wise relative to their Chl partners: the fluctuations are of the order of 0.5 Å or smaller (*conf*., Fig. 3a). Specifically, the central C_16_ = C_37_ bond is situated at (1.04 ± 0.52; −5.53 ± 0.41; −2.87 ± 0.30) Å for L1 and at (1.88 ± 0.45; −5.19 ± 0.34; −3.29 ± 0.29) Å for L2, which means that Lut 620 is slightly closer to the chlorin ring of its partner Chl *a*612 (*z* coordinate). At the same time both sites appear to be flexible enough to yield considerable orientational fluctuations. To quantify these fluctuations we introduce angles ${\phi }_{yx}$ and ${\phi }_{zx}$ (Fig. 3b,c). The former corresponds to the inclination of the backbone projection in the *xy* plane towards the *x* axis and partially relates to the rotation investigated in Fig. 2. The latter corresponds to the projection in the *xz* plane and describes the inclination of the Car towards its Chl. For the L1 site the mean values are ${\phi }_{yx}={(11.1\pm 4.2)}^{^\circ }$ and ${\phi }_{zx}={(6.8\pm 3.1)}^{^\circ }$. For the L2 site the mean values are ${\phi }_{yx}={(15.1\pm 3.5)}^{^\circ }$ and ${\phi }_{zx}={(3.0\pm 3.9)}^{^\circ }$. The corresponding distributions within the full trimer are shown in Fig. 3d,e. While Lut 621 is more inclined towards *y* axis than Lut 620, the opposite is true for the inclination towards the z axis. Interestingly, the latter trend is not observed directly within the crystal structure (see arrows in Fig. 3e), while Lut 621 in the MD trajectory relaxes toward markedly different orientation (Supplementary Fig. 4).

### Orientational dependence of Chl–Car coupling from probing configuration phase-space

We note that the rotation angle in Fig. 2, in its immediate vicinity around 0, effectively corresponds to the angle $\,{\phi }_{yx}$. The variation of ${\phi }_{yx}$ therefore means that modulation of the coupling is to be expected. However, we also note that comparable variation is present in the angle ${\phi }_{zx}$ as well. Lastly, the orientation of a rigid-body is fully described by three angles: in our case the rotation around the backbone axis of a Car is the third degree of freedom. All this points to the need to evaluate multiple Interaction Energy Surfaces (IES) in such a configuration phase-space. We calculated the IES for both sites (which represents the variation of coordinate in itself) varying the angles ${\phi }_{yx}$ and ${\phi }_{zx}$ and also the direction of the plane vector of the Luts. The latter dependence was found to be marginal (Supplementary Fig. 2).

**Figure 4:**
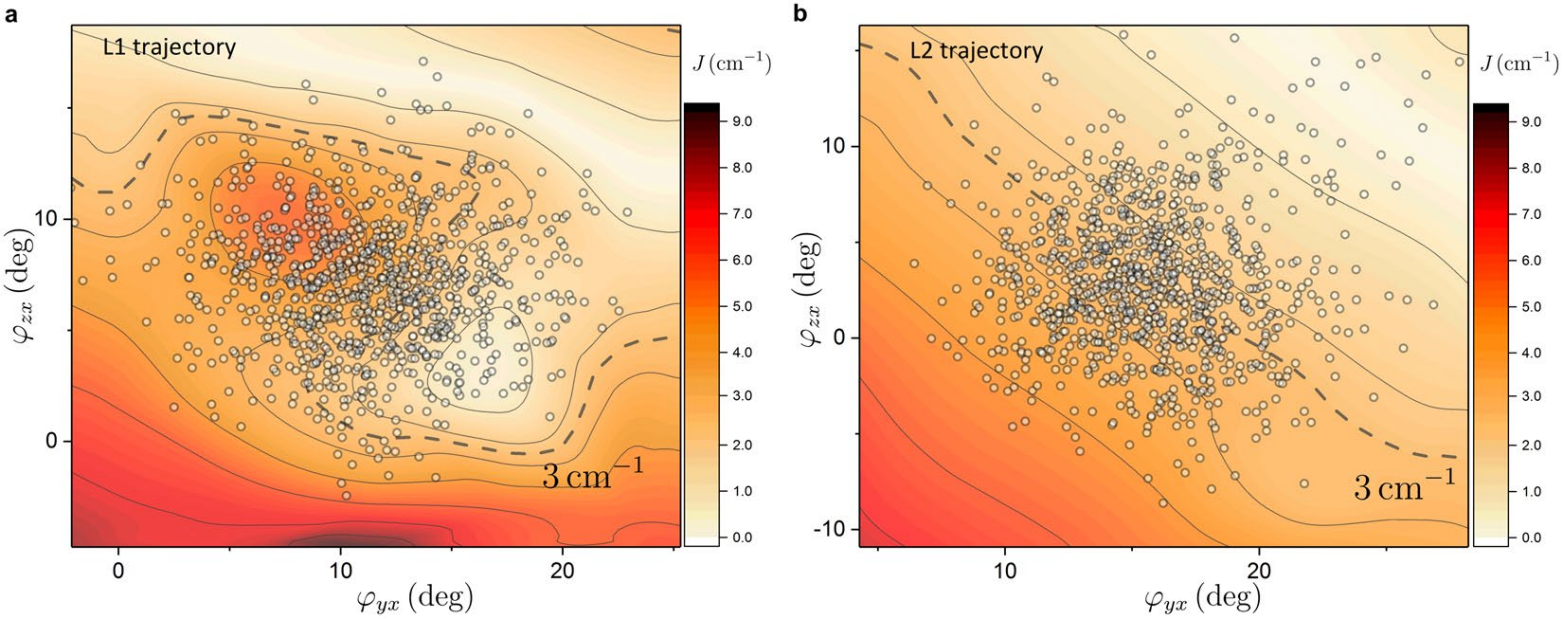
The Chl Q_y_ and Car S_1_ state interaction energy surfaces. (**a**,**b**) The IES (absolute values) are shown for the L1 and L2 sites, respectively. The white circles represent the actual values of the two angles from the MD trajectory. The dashed lines show the *J* = 3 cm^−1^ values for guidance. For the interpolation of the 41 TDC coupling values a 100 × 100 point grid was used.

The IES ${J}_{{\rm{L}}1/{\rm{L}}2}({\phi }_{yx},{\phi }_{zx})$ are shown in Fig. 4. Only absolute values of the couplings are shown, since the transfer rates are insensitive to the sign. The interpolated values of the couplings are shown along with the actual orientations from the MD trajectory (circles). The main feature of both surfaces is the decrease of the interaction energy going from smaller to larger angles, where the dependence passes the minimal *J* = 0 boundary. There are several minor local features too, especially at the L1 site. These features represent the fact that at such small distances the coupling is sensitive to even minute atomic differences (see Supporting Information). Interaction energy along ${\phi }_{yx}$ varies rather moderately and in line with the plateau-like dependence of Fig. 2. The variation in the ${\phi }_{zx}$ direction is stronger, and taken together they yield a substantial change in the interaction for relatively small deflections. The physiologically probable deflections can be inferred from the MD values of the orientation. While the average couplings are ${J}_{{\rm{L}}1}=3.6\pm 1.3$ cm^−1^ and ${J}_{{\rm{L}}2}=3.1\pm 0.9$ cm^−1^, they can increase almost twice or vanish within one trajectory. The angular dependence of the interaction energies for different displacements are shown and discussed in the Supplementary Information.

### Chl-to-Car excitation transfer, energy quenching and the functional role of the process

The calculated couplings provide insight into possible energy transfer processes in their own right, however, in order to appreciate their significance a full model of excitation relaxation is needed. Therefore, we firstly calculated the transfer rate of the excitation from the Q_y_ to the S_1_ state as a function of the angles ${\phi }_{yx}$ and $\,{\phi }_{zx}$. We used Förster resonance energy transfer (FRET) theory^34^, which is justified by the smallness of the coupling and the relatively large energy gap between the states^35^. Lut S_1_ energy was set to 14220 cm^−1^ as determined from the two-photon absorption data^36^ (see Supplementary Information), and the typical energy of Chl *a* Q_y_ state (14900 cm^−1^)^33^ was used. The angular dependence of the transfer rates on the coupling in Fig. 2 is shown to be very strong because of the $|J{|}^{2}$ factor (Supplementary Fig. 5). By contrast, the transfer rates mostly negligibly depend on the energy gap between Q_y_ and S_1_. This shows, that the uncertainty of the S_1_ energy is a minor factor in the details of such energy transfer.

**Figure 5:**
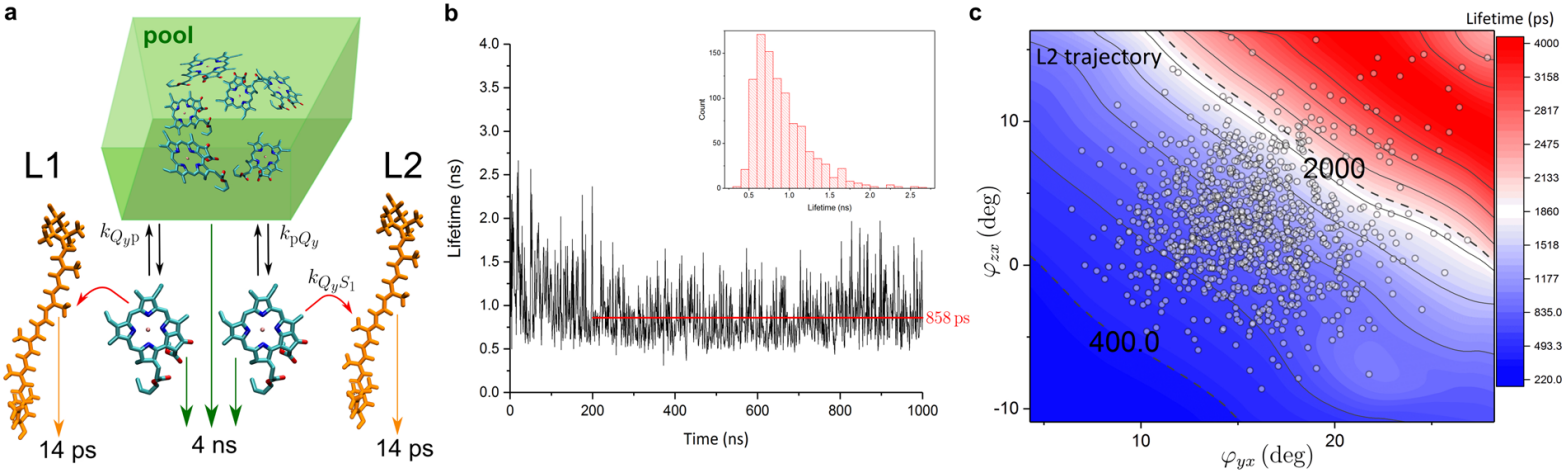
Lifetime of the Chl pool in the presence of Luts. (**a**) A pool of 6 Chl *a*’s (together with Chl *a*612 and Chl *a*603 representing the Chl *a* subsystem of an Lhca/Lhcb unit) is equilibrating with Chl *a*612/Chl *a*603 which in turn transfer excitation to their Car partners Lut 620/Lut 621. (**b**) The lifetime of the whole complex based on the L1/L2 pigment pair structures from the MD simulation. The inset shows the lifetime distribution based on the MD snapshots. (**c**) The lifetime of the complex as a function of the Car tilting angles, ${\tau }_{{\rm{complex}}}({\phi }_{yx},{\phi }_{zx})$, calculated for the coarse-grained model **a** using the L2 site IES, ${J}_{{\rm{L}}2}({\phi }_{yx},{\phi }_{zx})$, for both sites because of the smoothness of this particular IES. The dashed lines indicate the typical time-scales of the light-harvesting regime in the membrane (2 ns^37^) and the quenched regime under high-light conditions (~400 ps^38^). The circles represent the actual tilt angles for Lut 621 from the MD trajectory.

The calculated transfer rates demonstrate how the protein scaffold operates on the level of individual Chl–Car pairs, avoiding orientations that would facilitate excitation quenching via S_1_ state (Supplementary Fig. 5). Now we look at the role of such transfer in the overall biological functioning of the entire LHC sub-unit. In order to illustrate how transfer to S_1_ affects the excitation evolution within one Lhcb/Lhca complex under the conditions of closed RCs, we consider a coarse-grained, purely kinetic model, summarized in Fig. 5a. We partition the Chl *a* sub-population within a single LHC into the pool (6 Chl *a’*s; green block in Fig. 5a) and separate Chl *a*612 and Chl *a*603 (L1 and L2 sites, accordingly). All the Chl *b*’s are assumed to instantly populate the Chl *a* pool, which is lower in energy (80% of excitation transferred in less than 1 ps^16^). The pool transfers the excitation towards Chls *a*612/603 with the rate ${k}_{{{\rm{pQ}}}_{y}}$. The excitation leaves the singled-out Chls either back to the pool (rate ${k}_{{{\rm{Q}}}_{y}{\rm{p}}}$) or to their Car partners (the calculated FRET rates ${k}_{{{\rm{Q}}}_{y}{{\rm{S}}}_{1}}$). All Chls have the lifetime of 4 ns^37^, while the lifetime of Lut S_1_ state is considered 14 ps^22^. The robustness of this model with respect to parameter variation is discussed in the Supplementary Information.

Having calculated the IES, we map the coupling values onto particular instances of the L1/L2 pair orientations from the MD trajectory. The obtained coupling trajectory is then converted into the rates, which in turn are used within the coarse-grained model. The resultant values of the net lifetime of the whole complex, ${\tau }_{{\rm{complex}}}$, are shown in Fig. 5b. The transient dynamics (first ~200 ns) can be observed, where the initial lifetime of 1.5 ns drops to the subsequent average value of 0.86 ns. Such a sub-nanosecond lifetime has been observed for quenched LHCII crystals^39^, which is a sign that the MD trajectory is within a local minimum not too far from the crystal structure used as the starting point.

We further employ the coarse-grained model to see the effect of alternative Chl–Car orientations on the lifetime ${\tau }_{{\rm{complex}}}$. To that end we use the full IES of the L2 site (Fig. 4c) for both sites, because of the smoothness of this particular IES as opposed to the detailed IES of the L1 site (Fig. 4b). The resulting ${\tau }_{{\rm{complex}}}$ map is shown in Fig. 5c. Even though a number of approximations have been invoked, the result provides us with several valuable insights. Naturally, the diagonal dependence of the interaction energy translates into the diagonal dependence of the net lifetime. The strong coupling at small angles translates into short lifetime of the complex (lower left corner) and *vice versa* for the large angles (upper right corner). Again the strong dependence of FRET rates on the coupling leads to profound change of the lifetime upon slight changes in the angles. The red stripe, corresponding to the intrinsic Chl*a* lifetime of 4 ns, is separated from the light-harvesting configuration in the membrane (${\tau }_{{\rm{complex}}}\approx 2\,{\rm{ns}}$ ^37^) by just 5°−10°. A tilt reducing the angles by $\sim {5}^{^\circ }$ can bring the lifetime to the domain of values typical for the LHCII crystals (~1 ns), as mentioned previously. A further tilt of 5°−10° could bring the lifetime to as low as 400 ps, a characteristic value of LHCII lifetime under the NPQ conditions^38^. This means that, at least in principle, the re-orientation of the transition densities of Luts could be one of the key ingredients in switching from the light-harvesting state into the NPQ mode of operation within LHCs.

## Discussion

In principle, there are 6 explicit degrees of freedom in placing a Car molecule with respect to Chl: 3 coordinates to displace the molecular center and 3 angles to orient the molecule at a given position. Additionally, there are the “intrinsic” degrees of freedom that describe the actual molecular shape governed both by the chemical structure and the coordinating residues of the apoprotein. As it turns out, in such a vast phase-space of possible configurations, nature appears to be consistently restrictive (Fig. 1e,f). The benefit of close co-facial pairing of certain Chls and Cars has been rationalized in terms of efficient triplet quenching^40,41^. However, that alone does not explain the restrictive binding and its repetition throughout the variety of LHCs. Furthermore, having a pigment of such a short lifetime as Cars^22^ poses a threat to the excitation energy storage within the antenna before any subsequent transfer to the RCs can take place. Our coupling calculations, spanning the orientation phase-space simultaneously in several directions, revealed that the specific mutual orientation actually corresponds to the minimal resonance interaction between the lowest-lying singlet states of the two pigments (Fig. 2). This enables the photosystems to benefit from all the functionality of Cars (structural, light-harvesting and protective roles^22,42^) without depleting the system of energy.

On the other hand, the fact that Cars induce observable energy dissipation, even with the suppressed interaction, cannot be ignored (Fig. 5c). Even the modest couplings yield excitation quenching, the only question being whether the Luts in LHCII are only minor quenchers, or are they important enough to be the major agents of the NPQ mechanism? The ${|J|}^{2}$ factor within the FRET rate implies a very strong LHC lifetime dependence on the Chl–Car coupling, making the ensuing dynamics extremely susceptible to both the precise inter-pigment configuration and the intra-pigment transition density distribution. Increasingly, the sensitivity of this pathway is being discussed in the context of NPQ mechanism. Several recent studies cite the Car S_1_ state as the quencher of excess energy proposing that this pathway is modulated by *some* relative movement of the Chls and Cars^43–45^. Our MD simulations, in conjunction with the coupling calculations, provide insight into how specific variations of these configurational degrees of freedom play a physiological role. There are three major aspects regarding what can be learnt from the presented results.

### Position

There are arguments regarding Cars’ (non)involvement in NPQ that relate to the mutual Chl–Car positioning. One group of such arguments claims that no translational movement is possible within as tightly bound a scaffold as a protein, meaning there is simply not enough room for Cars to act as NPQ “switches”^46^. There is, however, another group of arguments anticipating very specific movements as switches^45,47^. The MD simulation favors the former arguments, because the position fluctuations are confined within ~1 Å. Of course, the simulated dynamics represent thermal fluctuations, not abrupt conformational changes, yet the coupling dependence on distance (see Supplementary Information) points to drastic displacements required for actual switching.

### Orientation

**Figure 6:**
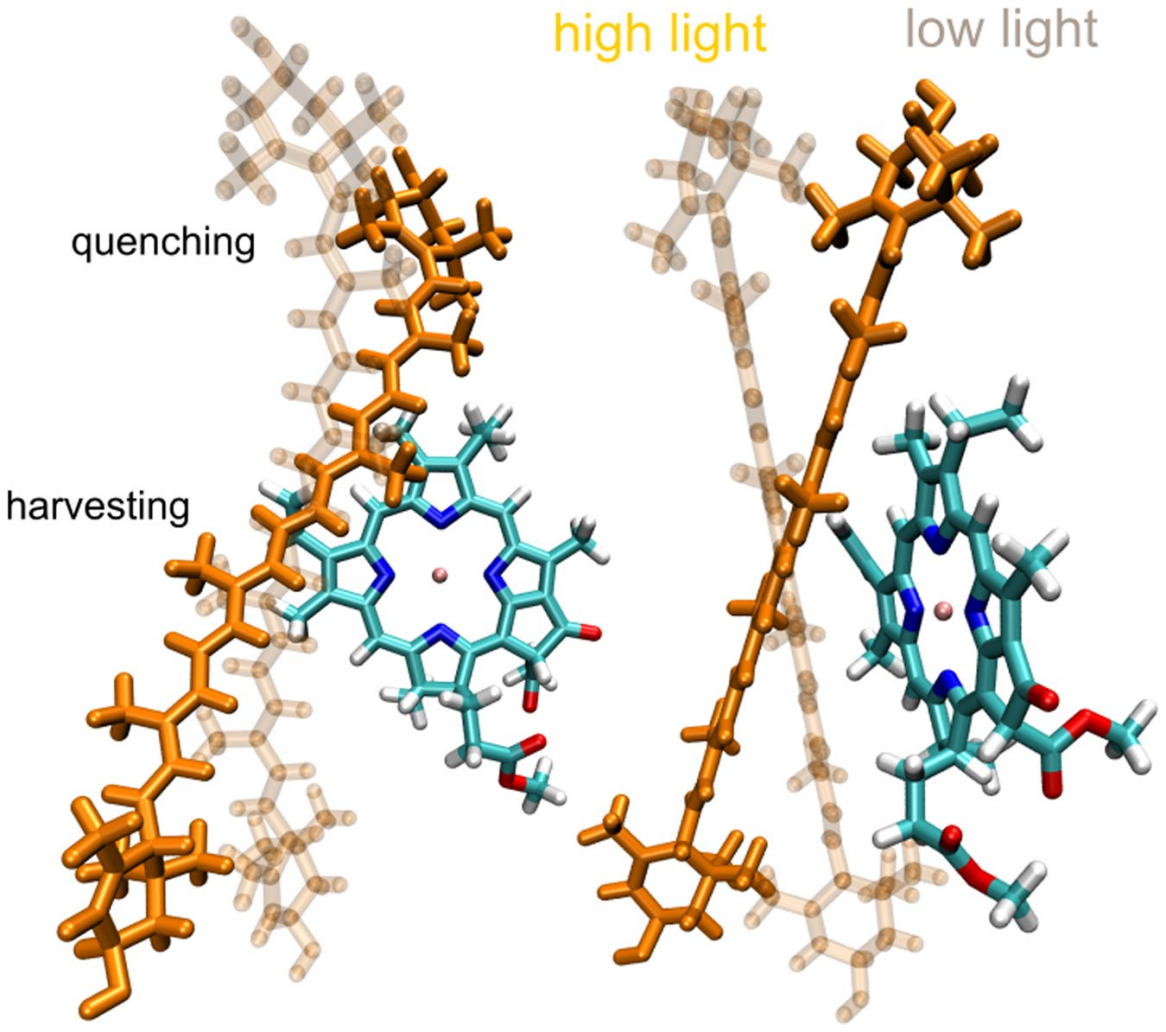
Representative structures of the boundary regimes of the excitation density control. A tilt of Luts with respect to Chls by less than 20° is sufficient to switch from the excitation energy preserving/light-harvesting mode (full orange structures) to the highly dissipative/quenching mode (transparent structures). The shown Lut structures correspond to the marginal angular values of Fig. 5c: the lower left corner (5.7°−9.2°, transparent) and the upper right corner $(({26.4}^{^\circ },{15.1}^{^\circ });\,J\approx 0;\,{\rm{full}}\,{\rm{color}})$.

As opposed to the distance, a wide angular distribution of the Chl with respect to the Car conjugated backbone is supported by the protein (Fig. 2d,e). More importantly, even moderate tilts are sufficient for a substantial change in the interaction, hence the sensitivity of the LHC excitation lifetime to the fluctuations of the mutual Chl–Car configuration within the MD trajectory. Such sensitivity in turn implies that the functional state of an LHC unit can be easily shifted towards a state that markedly decreases the excitation lifetime (Fig. 5c), supporting some earlier proposed NPQ mechanisms^48^. The physical orientations capable of inducing or preventing the dissipative regime in the whole LHC are shown in Fig. 6.

### Distortions

One common argument is that the Car S_1_ state may become “more allowed” in the face of protein-induced distortions away from planarity. This is an aspect disregarded in our coupling calculations (*conf*., Supplementary Fig. 3). While some deformations are present within the MD results, they are beyond the current computational capabilities due to complicated character of the S_1_ state combined with the system size^25^. However, even under such circumstances several reasonable and important observations can be made. There are two types of quantitative changes possibly induced by the deformation: a shift in energy and a change in the dipole moment. The former change appears to be irrelevant in the overall transfer because of smallness of the resonance interaction and strong influence of the environment (see discussion in the Supplementary Information). This additionally rules out any gearshift-type mechanism of NPQ which relies on closing the Chl–Car energy gap as a switch^23^. Interestingly, this indirectly points to the non-uniqueness of L1 site as a quencher in LHCII and CP29, which it was proposed to be based on the association with the lowest energy Chl cluster (Chl *a*610/611/612)^33^. The possible change in the dipole moment is more difficult to account for, but the trend can be named with certainty: due to deformation and the ensuing admixture of one-electron configurations into the S_1_ state (effective mixing with dipole-allowed state S_2_) its dipole moment can only increase^49^, thus increasing the coupling. At the same time such increase must be very well bound, for there is no spectroscopic evidence of the S_1_ state opening up upon deformation (*e.g*., heavily bent Neo in the N1 pocket does not produce S_1_ optical signal). But we agree that the geometry aspect is of paramount importance when dealing with the Car S_1_ state and needs further clarification, which would come with the development of more suitable quantum chemical methods. Lastly, there are qualitative geometrical changes associated with the head-group rotation, however, their quenching role is precluded by the virtual absence of transition density in these groups (Supplementary Fig. 1b,c).

In conclusion, we have demonstrated that the positioning of Cars within the green lineage eukaryotic antenna complexes is governed by the principle of minimal resonance interaction between Car S_1_ and spatially closest Chl Q_y_ states. This explains the remarkably regular Car binding maintained within homologous sites. Despite being minimal, the coupling is sensitive to even slight deviations of mutual pigment orientation. We demonstrate that a small increase in coupling translates into significant excitation quenching of the whole LHC unit. Therefore minor adjustments of Car orientation toward Chl sustained by the protein scaffold are sufficient for the transitions between light-harvesting and photoprotective (quenched) global states. The repetition of the Chl–Car configuration also implies that multiple NPQ sites are possible, not just within the major antenna. The obtained slow transfer-to-trap rates ensure that quenching does not compete with the open RCs and that the quencher is only significant once the RCs close. Such a concept of quenching has been termed “economic photoprotection”^50^. Lastly, the results show that further steered-MD studies provide a viable path for pinpointing the precise transitions between the harvesting/quenched configurations under *in vivo* conditions.

## Methods

### Electronic structure and coupling calculations

Ground state geometries of the molecules were optimized using density-functional theory (B3LYP functional) as implemented in Gaussian09 package^51^. The lowest excited singlet states of the vacuum-optimized pigments were calculated by a full Configuration Interaction calculation within a Complete Active Space using the semi-empirical AM1 Hamiltonian (AM1-CAS-CI) as implemented in the package MOPAC2016^52^. This methodology was benchmarked for Cars by Kusumoto *et al*.^53^ Using the obtained wave-functions we calculated the transition densities (custom code^54^) within the TDC famework^32^:

$$M({x}_{1},{y}_{1},{z}_{1})\approx {\int }_{{x}_{1}}^{{x}_{1}+\delta x}{\int }_{{y}_{1}}^{{y}_{1}+\delta y}{\int }_{{z}_{1}}^{{z}_{1}+\delta z}{\rm{d}}x{\rm{d}}y{\rm{d}}z\,{{\Psi }}_{g}{{\Psi }}_{e}^{\ast },$$

where ${\Psi }_{g}$ and ${\Psi }_{e}^{\ast }$ are the ground and excited state wave-functions, and $\delta x,\delta y,\delta z$ define the grid size of the cube. The electronic coupling (Coulombic part only) was calculated as $J=\frac{{e}^{2}}{4\pi \varepsilon {\varepsilon }_{0}}\sum _{i,j}{M}_{m}(i){M}_{n}(j)/|\mathop{{r}_{i}}\limits^{\rightharpoonup }-\mathop{{r}_{j}}\limits^{\rightharpoonup }|,$ where Chl and Lut transition dipole moments were re-scaled to 4.49 D^55^ and 0.767 D^25^, respectively.

### Molecular dynamics simulation

The high resolution X-ray crystal structure of LHCII from spinach^7^ (PDB: 1RWT) was used for MD simulation. We selected the trimer of chains C, H, and E. A DOPC (1,2-dioleoyl-sn-glycero-3-phosphocholine) bilayer membrane was generated by the CHARMM-GUI^56^ with 450 lipids in each layer. The membrane was generated in a rectangular box with upper and lower water layers containing 37 water molecules per lipid molecule. LHCII was pre-equilibrated before placing into the lipid bilayer: a LHCII shape cavity was generated at the center of the membrane by removing the lipids that were closer than 1.5 Å from the complex. Then, the LHCII complex was inserted into the pore according to the suggested orientation of protein membrane database^57^. The MD of LHCII embedded in the membrane was performed following the protocol described by Ogata and coworkers^58^. MD runs were performed with the Amber14 suite^59^. The Amber ff14SB force field was used to describe the protein^60^. All carotenoids were modelled by an *ad hoc* force field described in Prandi *et al*.^61^ Chls *a* were modelled with the set of parameters reported in Ceccarelli *et al*.^62^ and modified by Zhang and coauthors^63^; Chls *b* were described with the same set of parameters of Chls *a* except for the aldehyde group on porphyrin ring, taking parameters from the General Amber Force Field^64^. The DOPC membrane was described with the Lipid14 forcefield^65^. Since this force field for lipid does not contain parameters for the internal DPPG molecule, the previous version of the force field (Lipid11^66^) was used for it. Water molecules were described through the TIP3P model^67^, and ionic parameters were taken from Joung and Cheatham^68^. The full protocol is given in the Supporting Information.

### Resonance transfer rates

The FRET rates between pigments *m* and *n* are given by^69^

$${k}_{mn}=2{|{J}_{mn}|}^{2}{\rm{Re}}{\int }_{0}^{\infty }{A}_{m}(t){{F}_{n}}^{\ast }(t){\rm{d}}t,,$$

where ${J}_{mn}$ is the inter-pigment coupling and $A(t),F(t)$ are the acceptor absorption and donor fluorescence time-domain response functions, related to the corresponding spectra via the Fourier transform. Spectral information for Chl *a* is reported by Renger *et al*.^70^ while the corresponding parameters for Lut were extracted from the two-photon absorption data by Walla *et al*.^36^ as detailed in the Supporting Information.

### Coarse-grained model simulation

The evolution of the coarse-grained model of five sites (pool and four pigments) is governed by a Master equation, which in turn is fully described by the matrix of inter-site transfer and on-site decay rates. The pool-to-Chl *a*612/603 and the reverse rates, ${k}_{{{\rm{pQ}}}_{y}}$ and ${k}_{{{\rm{Q}}}_{y}{\rm{p}}}$, are related by the entropic factor: ${k}_{{{\rm{pQ}}}_{y}}={k}_{{{\rm{Q}}}_{y}{\rm{p}}}/6\equiv k/6$, which simply accounts for the fact that transfer from *n* sites to one particular site is *n* time less likely than the reverse; we used a characteristic value ${k}^{-1}=1\,{\rm{ps}}$. The lifetime of the complex ${\tau }_{{\rm{complx}}.}$ is then directly related to the eigenvalues of the matrix, as given in the Supporting Information along with the discussion of the possible parameter variation.

### Data availability

All the couplings, rates, simulation data and pigment pair structures are available upon request to the corresponding author.

## Electronic supplementary material

Supplementary Information
