# Supplementary material for: Fine control of chlorophyll-carotenoid interactions defines the functionality of light-harvesting proteins in plants
Source: Sci Rep. 2017 Oct 24;7:13956. doi: 10.1038/s41598-017-13720-6 (PMC5655323; doi:10.1038/s41598-017-13720-6)
Supplement: Supplementary file 1 — Supplementary Information [file 41598_2017_13720_MOESM1_ESM.pdf]

Supplementary Information to the article

Fine control of chlorophyll-carotenoid interactions defines the functionality of light-harvesting proteins in plants

Vytautas Balevičius Jr.[1], **Kieran F. Fox**[1], **William P. Bricker**[2], **Sandro Jurinovich**[3], **Ingrid G. Prandi**[4,5], **Benedetta Mennucci**[3], **Christopher D. P. Duffy**[1,*]

[1]*School of Biological and Chemical Sciences, Queen Mary University of London, London E1 4NS, UK*

[2]*Department of Biological Engineering, Massachusetts Institute of Technology, Cambridge, MA 02139, USA*

[3]*Dipartimento di Chimica e Chimica Industriale, University of Pisa, Via G. Moruzzi 13, Pisa, 56124 Italy*

[4]*Department of Chemistry, Federal University of Lavras, 37200-000, Lavras, Brazil*

[5]*Laboratory of Molecular Modeling Applied to the Chemical and Biological Defense, Military Institute of Engineering, Praça Gen. Tibúrcio, 80, Rio de Janeiro, Brazil*

[*]Correspondence: c.duffy@qmul.ac.uk

In this Supplementary Information, we provide computational details of the quantum chemical calculation of the pigment transition densities and inter-pigment coupling, subsequent Förster Resonance Energy Transfer (FRET) rates and the Light-Harvesting Complex (LHC) lifetime from the coarse-grained model of LHCII antenna. The description of Molecular Dynamics (MD) simulation is also given.

1 Quantum chemistry of the relevant pigment molecules

1.1 Lowest singlet excited states and transition densities.

Lutein (Lut) and chlorophyll *a* (Chla) molecules were prepared for all subsequent calculations by vacuum-optimization using DFT (B3LYP functional) as implemented in Gaussian09 package[1]. Chla phytol tail was truncated and methylated just after the ester group as the remaining moiety is known not to possess any transition density associated with the Q_y state (also, see further).

The lowest excited singlet states of the planar pigments were calculated by a full Configuration Interaction calculation within a Complete Active Space, using the semi-empirical AM1 Hamiltonian (AM1-CAS-CI) as implemented in the package MOPAC2016[2]. This method has been thoroughly tested by Kusumoto *et al.*[3] and shown to be consistent with more expensive methods. The method gives the lowest excited singlet state of Lut that is contributed 57.2% by double-excitation (2x HOMO-LUMO) configurations of the CI expansion. The lowest Chla excited singlet state is 81.7% HOMO-LUMO excitation. Calculated properties of the two molecules are summarized in Supplementary Table 1.

Supplementary Table 1 | Energies and dipole moments of lowest Lut and Chla singlet states.

State	Energy (calculated) (cm^{-1})	Energy (measured) (cm^{-1})	Dipole moment (calculated) (D)	Dipole moment (measured) (D)
Chla Q_y	16,700	14,900[4]	4.20	4.49-4.69[5]
Lut S_1	20,753	14,220[a]	0.49	---[a]

[a]Direct one-photon absorption of the S_1 state is not observable. Energy for Lut S_1 can be inferred indirectly, *e.g.*, via two-photon absorption[6] and subsequent modelling as described in the 3.1 Section.

For subsequent coupling calculations, the transition density distribution cloud was represent by Transition Density Cubes (TDCs) using custom code [7]. The TDCs are a discretization of the continuous transition density onto a finite 3D grid of transition charge elements (cubes):

$$M(x_1, y_1, z_1) \approx \int_{x_1}^{x_1+\delta x} \int_{y_1}^{y_1+\delta y} \int_{z_1}^{z_1+\delta z} \mathrm{d}x\mathrm{d}y\mathrm{d}z \, \Psi_g \Psi_e^* \, , \tag{1}$$

where Ψ_g and Ψ_e^* are the ground and excited state wave-functions, respectively, and $\delta x, \delta y, \delta z$ define the grid size of the cube. The resultant transition densities are shown in **Supplementary Figure 1**. The TDCs are individually re-calculated for each pigment re-orientation in the study.

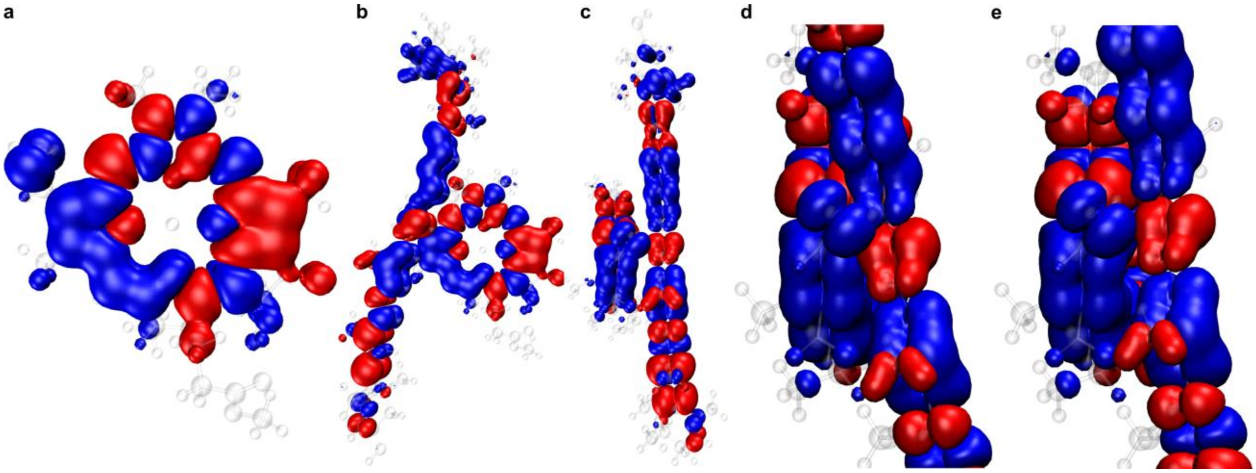

Supplementary Figure 1 | Transition densities of Lut and Chla. (**a**) Transition density of just Chla Q_y state; there is no transition density in the phytol tail. Color coding (red and blue) is used to highlight the opposite sign of the transition charges in the corresponding regions. (**b,c**) Transition densities of both Lut and Chla in the mutual arrangement within LHCII (front and side views, respectively). Transition densities only marginally extend to the end groups of Lut. Note that the transition density of Lut is several orders of magnitude smaller than that of Chla, and appears similar here only because of different scaling. (**d,e**) The zoom-in of the region of closest proximity to demonstrate the subtle difference of the L1 (**d**) and L2 (**e**) sites (the transition density cloud is essentially the same, only the orientations differ). The VMD package[8] was used for plotting molecular structures here and in the subsequent Supplementary Figures.

1.2 Couplings form the transition density cube method.

We approximate the electronic coupling by its Coulombic part, which was calculated using the obtained TDCs via pairwise interaction between pigments *m* and *n*:

$$J^{\mathrm{TDC}} = \frac{e^2}{4\pi\varepsilon\varepsilon_0} \sum_{\substack{i\in m \\ j\in n}} \frac{M_m(i) M_n(j)}{\left|\vec{r_i} - \vec{r_j}\right|} . \tag{2}$$

The coupling calculation is carried out using the code by Krueger *et al*.[9]. To gauge the calculated coupling, we re-scaled the transition dipole moments (TDM) of the pigments. The Chla Q_y TDM was re-scaled to 4.49 D (a recommended value given by Knox and Springs[5]), while the TDM of Lut S_1 (importantly, non-zero) was re-scaled to 0.767 D (a value reported in the literature reviewing more sophisticated approaches[10]).

We note that at such small distances as considered in this study the coupling is sensitive to the precise intermolecular arrangement, where particular atom groups of one molecule appear in the immediate vicinity of the neighboring molecule. The fine features in the Interaction Energy Surface (IES) of the L1 site (**Fig. 4a** of the main text), in comparison to the monotonic surface of L2 site reflect this sensitivity. The difference in the transition density distribution of the two sites is shown in **Supplementary Fig. 1d,e**, where Lut in site L1 is appears notably closer to Chla. However, it is also possible that such sensitivity to the details is overly expressed in the TDC method.

1.3 Coupling dependence on co-faciality of the pigment pair.

To study which degrees of freedom have largest influence over the Chla–Lut interaction, we investigated, among others, the rotation of Lut around its backbone axis (azimuthal rotation in **Supplementary Figure 2a**). This angle effectively describes the facing of the polyene plane (or co-faciality of Lut and Chla).

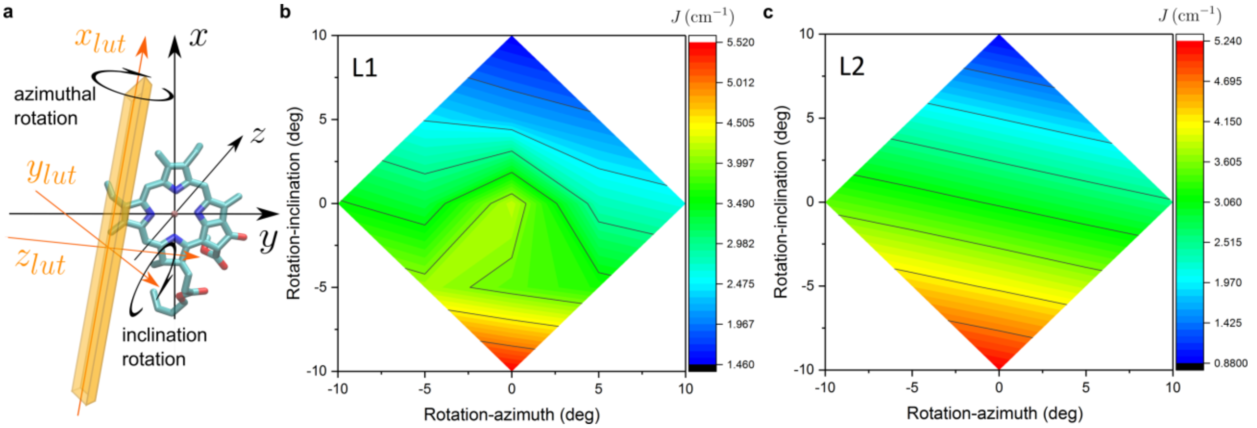

Supplementary Figure 2 | Chl–Lut interaction energy dependence on the planar tilting of Lut. (**a**) Internal coordinates of Lut: x_{lut} axis along the backbone, z_{lut} axis perpendicular to the molecular plane and towards Chla, y_{lut} axis perpendicular to x_{lut} and z_{lut} (within molecular plane). Azimuthal rotation around x_{lut} defines the "co-faciality" of Lut towards Chla; inclination towards Chla is governed by rotation around y_{lut}. (**b,c**) Interaction Energy Surfaces (IES) corresponding to azimuthal- and inclination-rotations of Lut within its reference frame at L1 (**b**) and L2 (**c**) sites, accordingly. The plots are based on coupling values at 13 orientations, combining rotations of ±5° and ±10° in both directions.

As can be seen from **Supplementary Figure 2b,c** the effect of azimuthal rotations is almost negligible, especially compared to the change of Lut inclination towards or away from Chla. We note that in the Chla reference frame (the reference frame of the main article) the inclination rotation is effectively decomposed into the changes of $(\varphi_{yx}, \varphi_{zx})$ angles. Hence, we have excluded the planar tilting from the considerations of the main article.

2 Molecular Dynamics simulation

2.1 Simulation procedure and parameters.

Crystal structure preparation. The high resolution X-ray crystal structure of LHCII from spinach (PDB: 1RWT [11]) was used for MD simulation. We selected the trimer made of chains C, H, and E. The structure was cleaned by retaining the protein, the pigments, the 1,2-dipalmitoyl-*sn*-glycero-3-phosphoglycerol (DPPG) lipid molecule and the interstitial water molecules. The N-terminus chains are not completely resolved in the crystal structure and the first 13 residues are missing, therefore we capped Ser-14 with an acetyl group. Following the Poisson-Boltzmann calculations by Müh *et al.*[4] , all the titratable amino acids were considered in their standard protonation state at physiologic pH, except for His-120, which was considered protonated due to its interaction with Asp-111. All the other histidine residues axially coordinating the Mg atom of Chls were considered in δ conformation. Missing heavy atoms in Chls *a*604, *b*605, *b*606 and *a*614 phytol tails were added according to the Chl's structure by manually adjusting the torsion angles to avoid structural clashes. Finally, the position of the added heavy atoms and all the hydrogen atoms were minimized with 500 steps of steepest descent followed by 500 steps of conjugate gradient.

Membrane equilibration. A DOPC (1,2-dioleoyl-sn-glycero-3-phosphocholine) bilayer membrane was generated by the CHARMM-Gui interface[12] with 450 lipids in each layer. The membrane was generated in a rectangular box with upper and lower water layers containing 37 water molecules per lipid molecule. The ionic strength was set at 0.1 M by randomly placing Na^+ and Cl^-. Before placing the complex into the lipid bilayer, a pre-equilibration of the solvated membrane alone was performed according to the following protocol: i) minimization of the whole system (5000 steps of steepest descent followed by 5000 step of conjugate gradient) with periodic boundary condition; ii) first heating run (5 ps from 0K to 100K) in the NVT ensemble, under positional harmonic constraints (10 KCal mol^{-1} $Å^{-2}$) on lipid molecules; iii) second heating run (100 ps from 100K to 300K) in the NPT ensemble by applying positional harmonic constraints (10 KCal mol^{-1} $Å^{-2}$) on lipid molecules; iv) membrane equilibration run (55 ns at 300K) in NPT ensemble without any constraint.

Insertion of the complex in the membrane. The atom coordinates taken from the last step of the membrane equilibration run were translated in order to put the centre of mass of the lipid membrane in the origin of the Cartesian system. A LHCII shape cavity was generated at the centre of the membrane by removing the lipids that were closer than 1.5 Å from the complex. Then, the LHCII complex was inserted into the pore according to the orientation suggested by the protein membrane (OPM) database[13]. The final system contains a total of 209728 atoms.

MD protocol. The molecular dynamics of the LHCII embedded in the membrane was performed following the protocol described by Ogata and coworkers[14] in the simulation of the PSII supercomplex:

1) *Three-step minimization*: i) minimization of all hydrogen atoms; ii) minimization of ions and non-crystal water molecules (upper and lower water layers added to the system); iii) all atoms minimization.

1. *Heating*: 30 ps of heating run up to 300K in NVT ensemble. The crystallographic structure (protein, pigments, DPPG, interstitial water molecules) was constrained by a harmonic potential with a 5 KCal mol^{-1} $Å^{-2}$ force constant.

2. *Equilibration*: The equilibration run was performed gradually releasing the harmonic constraints (from 5 to 0 KCal mol^{-1} $Å^{-2}$, reducing by -0.25 KCal mol^{-1} $Å^{-2}$ every 10 ps). This step was run in NPT ensemble with anisotropic pressure coupling. The 300 K temperature was controlled by the Langevin thermostat. When the force constraint became equal to zero, we completed the equilibration with an additional run of 2 ns without any constraint.

3. *Production*: 1µs of production run in the NPT ensemble at 300K was performed without any constraint. From this MD simulation, 1000 snapshots captured every 1 ns were used for the geometrical analysis (following Section 2.2) and QM calculations (Section 1.1).

Details of the MD simulations. All MD runs were performed with the Amber14 suite of programs[15], by using the GPU version of the code. An integration time step of 2 fs was used in combination with the SHAKE algorithm applied to the hydrogen atoms. Temperature and pressure were controlled by using the Langevin thermostat (collision frequency of 1.0 ps^{-1}) and the anisotropic Berendsen weak-coupling barostat (coupling constant of 1.0 ps^{-1}) respectively. Thanks to the new Lipid14 force field (see below for details), no additional superficial tension is needed[16]. The Particle Mesh Ewald method was used to treat the electrostatics, by applying a cut-off of 10 Å for long-range interactions.

Force Fields. The Amber ff14SB force field was used to describe the protein[17]. All carotenoids were modelled by an *ad hoc* force field described in Prandi *et al.*[18] Chlorophylls *a* were modelled with the set of parameters reported in Ceccarelli *et al.*[19] and modified by Zhang and coauthors[20]; chlorophylls *b* were described with the same set of parameters of chlorophylls *a* except for the aldehyde group on porphyrin ring, whose parameters was taken from the General Amber Force Field[21]. The DOPC membrane was described with the Lipid14 forcefield[16]. Since this force field for lipid does not contain parameters for the internal DPPG lipid molecule present in the crystal structure, the previous version of the force field (Lipid11) was used for DPPG[22]. Water molecules were described through the TIP3P model[23], and ions' parameters were taken from Joung and Cheatham[24].

2.2 Superimposition of the planar structures onto the structures from the MD trajectory.

In order to obtain most reliable results, we calculate the couplings using the vacuum-optimized (Section 1.1) "model" Lut and Chla molecules superimposed on the originals, rather than the actual structures from either crystallography or MD simulations, which have distorted bond structure. The superimposition is performed using pair fitting routine of the PyMOL software[25]. While the four nitrogens of the chlorin ring are used for pair fitting the Chlas, for Car the four backbone carbons having attached methyl groups and the four methyl carbons (8 atoms altogether) are used. The results of the procedure are shown in **Supplementary Figure 3** for a randomly selected snapshot of both L1 and L2 sites from the single monomer.

While Chla demonstrates nearly-perfect coincidence (except for the unimportant phytol tail), the MD Cars slightly deviate from the planar structures (more pronounced S-shape not only within the molecular plane, but also away from the plane) and have their β- and ε- end-groups free to rotate.

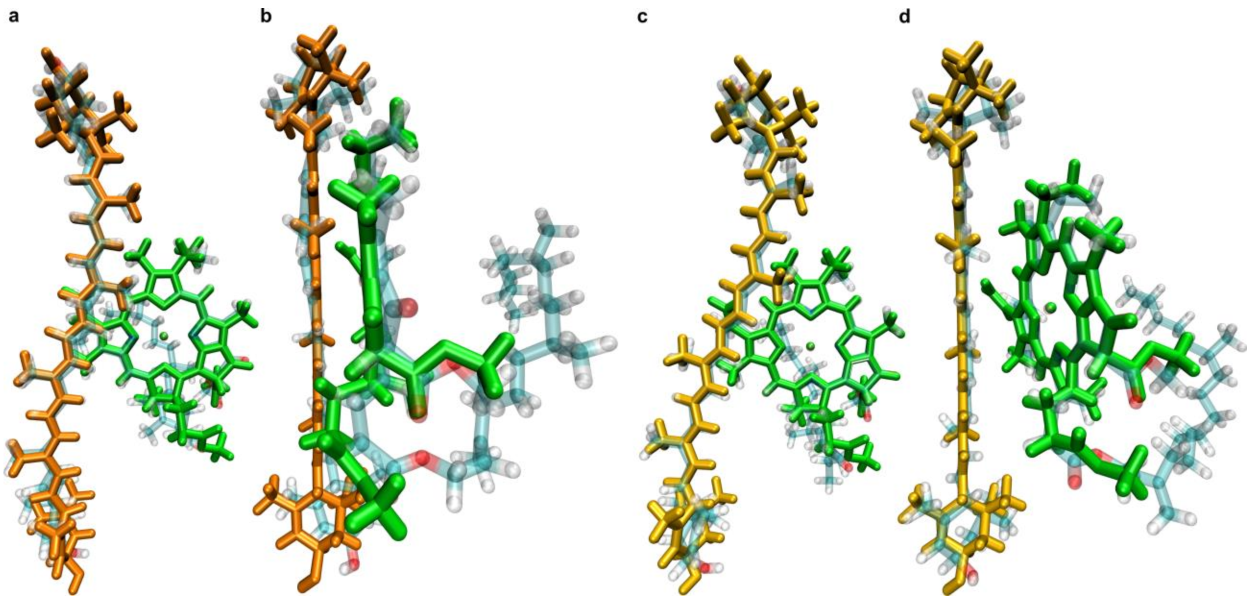

Supplementary Figure 3 | Comparison of the original MD snapshot structure with the planar optimized superimposed molecules. (**a-d**) A randomly selected MD snapshot structure (blue) with planar vacuum-optimized Lut (orange/yellow) and Chl*a* (green) molecules superimposed on it. The structures are shown for L1 (**a,b**) and L2 (**c,d**) sites, respectively. Front (**a,c**) and side (**b,d**) views with respect to Lut are shown.

2.3 Angular distribution among the L1/L2 sites and C/E/H monomers.

The distribution of inclination angles φ_{yx} and φ_{zx} (see the main text) shows some deviation from the normal distribution (*conf.*, **Figure 3d,e**). Therefore we analyzed several cuts of the distributions evaluating the temporal and monomeric differences.

Some initial transient behavior was identified within the trajectory. In **Supplementary Figure 4a-d** two subpopulations within C monomer are shown separately: the sub-200 ns values (red) and the subsequent values (blue). While for the L1 site both initial and subsequent values essentially overlap, a shift can be seen among the L2 site sub-distributions. We also show the corresponding angles determined for the two sites in the LHCII crystal structure (shown in black; PDB: 1RWT[11]). Interestingly, it appears that, while the L1 site relaxed values in the membrane roughly coincide with the distribution in the crystal structure, the L2 site crystal values coincide with the initial distribution, but not the subsequent one. This shift might indicate some reorganization within the membrane with respect to the crystal structure upon relaxation. The effect is observed to a similar or lesser degree in the other two monomers (not shown explicitly).

The distributions among all three monomers are shown in **Supplementary Fig. 4g-h**. The values of φ_{zx} roughly coincide within the whole trimer (**f** and **h**), while for φ_{yx}, H monomer is a slight outlier (**e** and **g**).

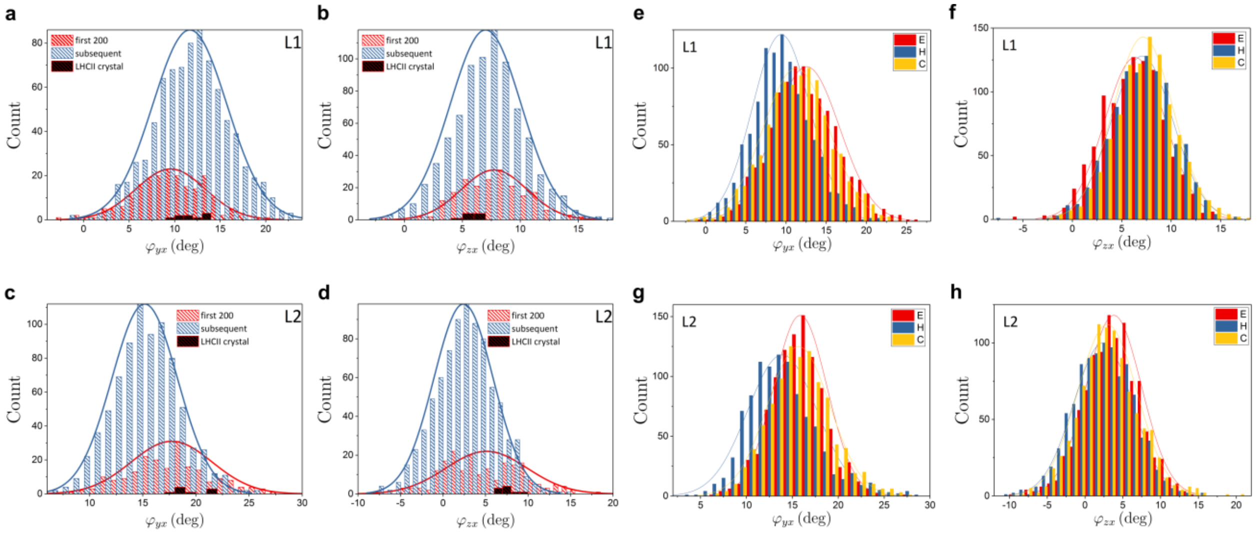

Supplementary Figure 4 | Comparison of the angular distribution among the sites and the monomers. (**a-d**) Distribution of the inclination angles within L1 and L2 sites of the C monomer. The sub-200 ns values are shown in red; the subsequent values are shown in blue. The full lines represent the normal distribution superimposed on the corresponding data. Additionally, the results for the LHCII crystal data (PDB: 1RWT[11]) are shown in black. In the latter case, the fitting of planar molecules over the crystal counterparts was done for each monomer of the three trimers captured in the crystal structure, hence the dispersion in the values. (**g-h**) Distribution of the two angles among the two sites within all three monomers of the LHCII trimer (C yellow; E red; H blue).

3 Resonance energy transfer calculations and quenching in the coarse-grained model

3.1 Förster resonance energy transfer (FRET) calculations and relevant parameters.

FRET rates between pigments *m* and *n* are given by

$$k_{mn} = 2|J_{mn}|^2 \mathrm{Re} \int_0^\infty A_m(t) F_n{}^*(t) \mathrm{d}t \,, \tag{3}$$

where J_{mn} is the resonance interaction between the pigment pair and

$$A_n(t) = \mathrm{e}^{-\frac{\mathrm{i}E_n t}{\hbar} - g_n(t)} \tag{4}$$

and

$$F_m(t) = \mathrm{e}^{-\mathrm{i}\left(\frac{E_m - 2\lambda_m}{\hbar}\right)t - g_m{}^*(t)} \tag{5}$$

are the acceptor absorption and donor fluorescence time-domain response functions, E_n and E_m are the molecular energies (measured energies in **Supplementary Table 1**), and $2\lambda_m$ is the reorganization energy (Stokes shift). The line-shape function is given by:

$$g_n(t) = \int_0^\infty \frac{\mathrm{d}\omega}{\pi\omega^2} C_n''(\omega) \left[\left(1 - \cos(\omega t)\right) \coth\left(\frac{\hbar\omega}{2k_B T}\right) + \mathrm{i}(\sin(\omega t) - \omega t) \right] \tag{6}$$

Here, $C_n''(\omega)$ denotes the spectral density. For the Chla, the response functions correspond to physical spectra defined by the spectral density suggested by Renger *et al.*[26]:

$$C_n''(\omega) = \frac{\pi S_0 \omega^5}{s_1 + s_2} \sum_{i=1}^{2} \frac{s_i}{7!\, 2\omega_i{}^4} e^{-\sqrt{\frac{\omega}{\omega_i}}} \tag{7}$$

where $S_0 = 0.5$, $s_1 = 0.8$, $s_2 = 0.5$, $\omega_1 = 0.56\ \mathrm{cm}^{-1}$ and $\omega_2 = 1.94\ \mathrm{cm}^{-1}$.

The carotenoid spectral density is generated to fit the two-photon absorption spectra by Walla *et al.*[6]. It is given by a decomposition into two terms for the underdamped modes (corresponding to two high-frequency carbon-carbon stretching modes) and one overdamped mode (to account for the rest of the thermal bath):

$$C''(\omega) = \sum_{i=1,2} 2\lambda_i \frac{\omega\omega_i^2\gamma_i}{(\omega^2 - \omega_i^2)^2 + \omega^2\gamma_i^2} + 2\lambda_0 \frac{\omega\gamma_0}{\omega^2 + \gamma_0^2}. \tag{8}$$

Here, the values of the double- and single-bond stretching frequencies[27] are $\omega_1 = 1500\ \ \mathrm{cm}^{-1}$ and $\omega_2 = 1150\ \ \mathrm{cm}^{-1}$, accordingly. The rest of the parameters are determined by fitting the two-photon absorption spectrum: $\lambda_1 = \lambda_2 = 900\ \mathrm{cm}^{-1}$, $\gamma_1 = \gamma_2 = 300$ fs for the underdamped modes; $\lambda_0 = 450\ \mathrm{cm}^{-1}$, $\gamma_0 = 53$ fs for the overdamped bath.

The measured transition energies are used: $14{,}900\ \ \mathrm{cm}^{-1}$ for Q_y state of Chla; $14{,}220\ \ \mathrm{cm}^{-1}$ for the S_1 state of Lut from the aforementioned fitting.

To examine the sensitivity of the FRET rates in the current setup to the energy gap between the pigments, we employ the inter-pigment coupling dependence given in **Fig. 2** of the main article (*i.e.*, the dependence of the interaction energy on the rotation of Chla around its *z* axis). In **Supplementary Figure 5** the results are shown for the main set of parameters (dark-green line) as well as for the limiting values (light-green band). As can be seen, even in the regions of fairly strong coupling the change of energy gap over 1000 cm^{-1} yields no significant effect. This is the joint effect of small resonance interaction and large reorganization energy of Lut.

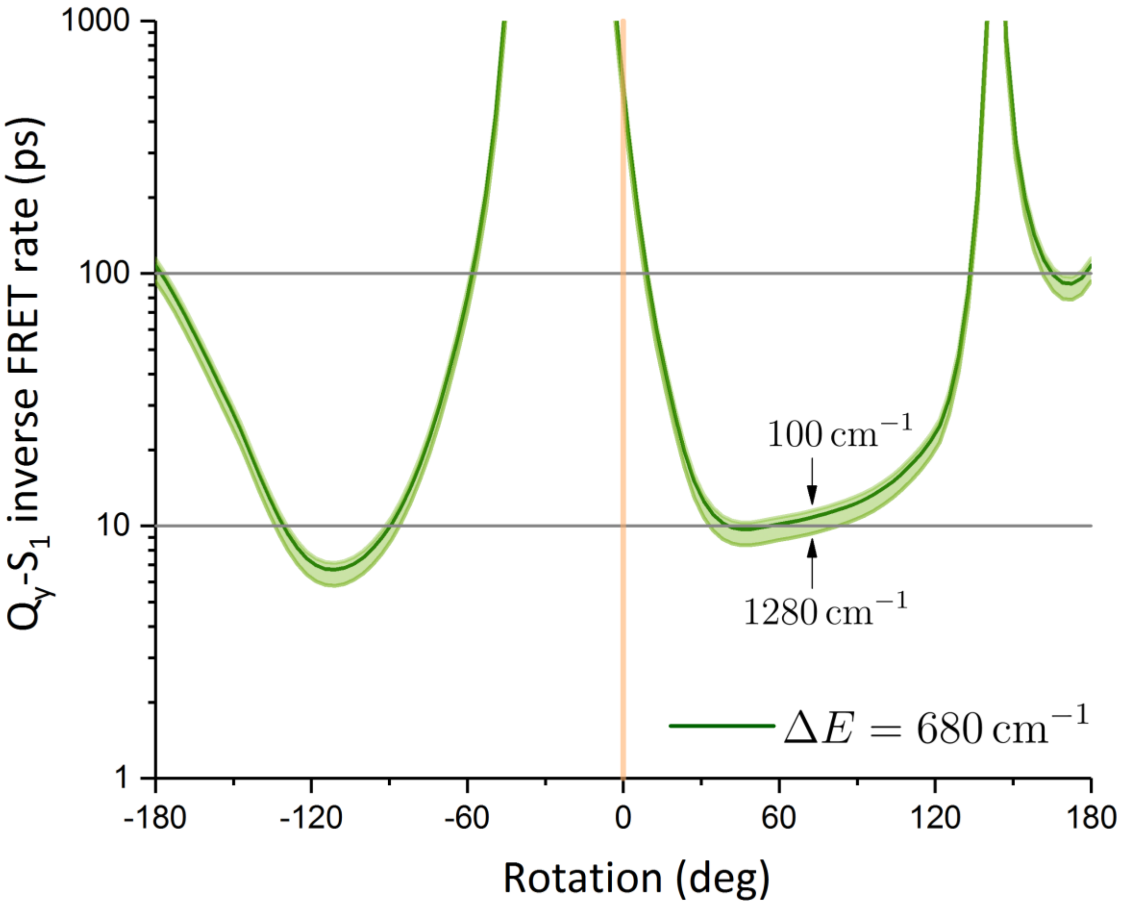

Supplementary Figure 5 |Chla-to-Car transfer times by FRET calculation. Values of the coupling are given in the main article (**Fig. 2**). The upper boundary corresponds to Chla Q_y state being just above the S_1 state, while the lower boundary corresponds to Q_y value for chlorophyll *b* (15,500 cm^{-1})[4].

The insensitivity to the energy gap has several important biological implications. Firstly, the dissipation-via-S_1 channel cannot be opened or closed in LHCs by modulating the energy gap. Secondly, there is no substantial difference between L1 and L2 sites, as sometimes is anticipated based on the fact Chla of L1 site belongs to the trio of lowest-energy Chls (so-called "terminal emitter")[4]. The dynamical arguments thus do not support the conjecture of L1 pair being the preferential quenching site. And last but not least, it is important to note that the established rate independence on energy gap actually provides the robustness and stability of the functioning the pairs within the fluctuating environments.

3.2 Coarse-grained kinetic model.

According to the coarse-grained model of an LHC monomeric unit five sites are defined: (1) the collective pool of 6 Chlas of an LHC (excluding the two L1/L2 Chlas); (2) L1 Chla; (3) L1 Lut; (4) L2 Chla; (5) L2 Lut. The excitation evolution of the $\boldsymbol{n} = (n_1, n_2, n_3, n_4, n_5)$ sites is described by a Master equation:

$$\frac{\mathrm{d}}{\mathrm{d}t}\boldsymbol{n}(t) = \boldsymbol{A}\boldsymbol{n}(t), \tag{9}$$

where **A** is the following matrix of rates:

$$\boldsymbol{A} = \begin{cases} -\kappa - \frac{k}{m} - \frac{k}{m} & k & 0 & k & 0; \\ \frac{k}{m} & -\kappa - k - k_{\mathrm{L1}} & 0 & 0 & 0; \\ 0 & k_{\mathrm{L1}} & -\gamma & 0 & 0; \\ \frac{k}{m} & 0 & 0 & -\kappa - k - k_{\mathrm{L2}} & 0; \\ 0 & 0 & 0 & k_{\mathrm{L2}} & -\gamma. \end{cases}$$

(10)

The rates of the matrix are defined as follows: $\kappa = (4\ \mathrm{ns})^{-1}$ is the decay rate of Chla in solution, $k = 1\ \mathrm{ps}^{-1}$ is an approximate effective rate of hopping from Chla to Chla, which is weighted for a pool-to-Chla transfer by an entropic factor $m = 6$, which is the number of pool (non-L1/L2) Chlas; $k_{\mathrm{L1/L2}}$ is the FRET rate from Chla to Lut in the corresponding L1/L2 site (back transfer is too slow to be considered), while $\gamma^{-1} = 14$ ps is the lifetime of Lut in solution.

The eigendecomposition of matrix **A**, given as $\boldsymbol{A} = \boldsymbol{C}\boldsymbol{\lambda}\boldsymbol{C}^{-1}$ (where $\boldsymbol{\lambda}$ is the vector of eigenvalues and **C** is the diagonalizing matrix), provides us with the solution to the Equation (9), and the lifetime of the whole complex is given by

$$\tau_{\mathrm{complx.}} = -Tr\{\boldsymbol{C}\boldsymbol{\lambda}^{-\mathbf{1}}\boldsymbol{C}^{-1}\boldsymbol{n}(0)\}$$

(11)

where *Tr* denotes a trace, and $\boldsymbol{n}(0) = \left(\frac{6}{8}, \frac{1}{8}, 0, \frac{1}{8}, 0\right)$ is the initial condition for the excitation of the LHC unit (we consider an instantaneous – direct or via Chl *b'*s – excitation of either of the Chla).

To check the model stability against a variation of the parameters (essentially, Lut lifetime and inter-Chla transfer times) we used the FRET rates corresponding to **Suppl. Fig. 5** (main energy gap value) for both sites. Results are shown in **Supplementary Figure 6**. As can be seen, within all the relevant net time-scales the coarse-grained model is insensitive to the differences between the possible parameters and the ones we used.

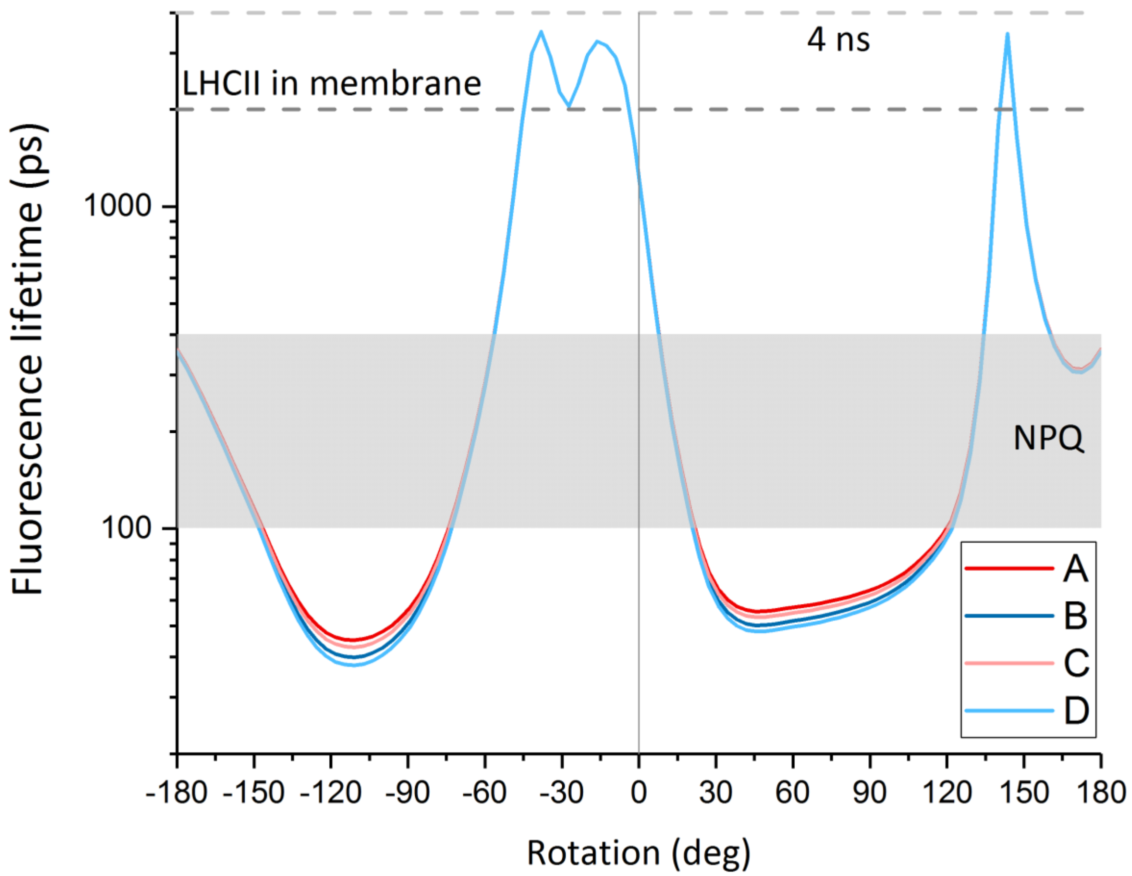

Supplementary Figure 6 | Dependence of the LHC fluorescence lifetime on the parameter variation. The four parameter sets are used: $k^{-1} = 0.5$ ps and $\gamma^{-1} = 20$ ps (A) or $\gamma^{-1} = 14$ ps (B); $k^{-1} = 1$ ps and $\gamma^{-1} = 20$ ps (C) or $\gamma^{-1} = 14$ ps (D; basic set). The grey shaded area represent the LHC lifetime values associated with the non-photochemical quenching (NPQ) regime[28], while typical lifetime of 2 ns is associated with the LHC within membrane[29].

3.3 Coupling and lifetime dependence on the inter-pigment distance

To fully explore the inter-pigment configuration phase-space, we have also studied the distance dependence of the coupling. To that end we have displaced Chla by a vector (-1 Å,+1 Å,+1 Å) from its original position within L1 site (for directions see main text **Fig. 1e,f**). This parallel shift effectively increased the inter-pigment distance by ~1.73 Å. The resultant IES is shown in **Supplementary Figure 7b**. The coupling is reduced and somewhat loses the directional sensitivity as compared to the original IES (**Supp. Fig. 7a**), however, it does not fall off directly to zero. As in the main text we translate this difference into the LHC lifetime maps (**Supp. Fig. 7c-e**): the original map from the main text is repeated (**c**), and the re-calculated IES (panel **b**) is used either for L1 site (**d**) or both sites (**e**). In the latter case the LHC lifetime approaches the 2 ns, however, the significant part of the map still covers sub-2-ns lifetimes.

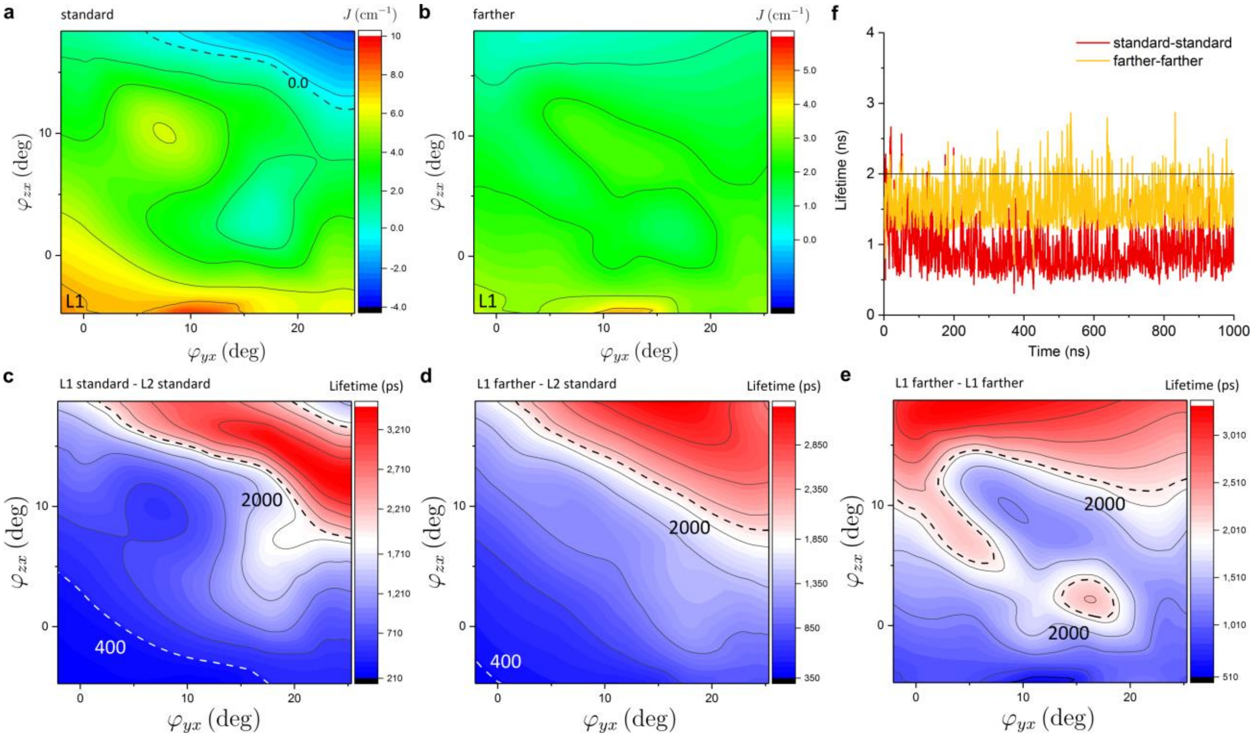

Supplementary Figure 7 | Coupling and complex lifetime dependence on inter-pigment distance. (**a**) IES of L1 site from the main article (**Figure 4a**). (**b**) The same IES of L1 site when Chla is moved away from Lut by 1 Å in each direction. (**c-e**) The net lifetimes of an LHC calculated for the coarse-grained model: using the IES of the main article (**c**); using the displaced IES **b** for L1 site, retaining the original IES for the L2 site (**d**); using the displaced IES **b** for both L1 and L2 sites (**e**). (**f**) The lifetime trajectory taking the Lut orientations from the MD simulation and using the standard lifetime map **c** (red line) or the displaced Chla maps **e** for both sites (yellow line).

To demonstrate the effect of both sites exploring the IES of displaced Chla (panel **b**), we plot the lifetime dependence on the orientations from the MD trajectory as shown in **Supp. Fig. 7f**. It appears, that moving Chla away from Lut even by 1.7 Å, which is both beyond the thermal fluctuations (conf., **Figure 3a**) and highly unlikely in such a rigid structure as a protein, still keeps the lifetime well beyond the lifetimes associated with the unquenched LHCs in the membrane[29].

References

1. Frisch, M.J. et al. Gaussian 09. (Gaussian, Inc., Wallingford, CT, USA, 2009).
2. Stewart, J.J.P. MOPAC2016. 16.175W edn (Stewart Computational Chemistry, 2016).
3. Kusumoto, T. et al. Femtosecond Transient Absorption Spectroscopic Study of a Carbonyl-Containing Carotenoid Analogue, 2-(all-trans-Retinylidene)-indan-1,3-dione. *J. Phys. Chem. A* **115**, 2110-2119 (2011).
4. Müh, F., Madjet, M.E.A. & Renger, T. Structure-Based Identification of Energy Sinks in Plant Light-Harvesting Complex II. *J. .Phys. Chem. B* **114**, 13517-13535 (2010).

5. Knox, R.S. & Spring, B.Q. Dipole strengths in the chlorophylls. *Photochem. Photobiol.* **77**, 497-501 (2003).
6. Walla, P.J., Linden, P.A., Ohta, K. & Fleming, G.R. Excited-state kinetics of the carotenoid S-1 state in LHC II and two-photon excitation spectra of lutein and beta-carotene in solution: Efficient car S-1 -> Chl electronic energy transfer via hot S-1 states? *J. Phys. Chem. A* **106**, 1909-1916 (2002).
7. Bricker, W.P. & Lo, C.S. Efficient Pathways of Excitation Energy Transfer from Delocalized S2 Excitons in the Peridinin-Chlorophyll a-Protein Complex. *J. Phys. Chem. B* **119**, 5755-5764 (2015).
8. Humphrey, W., Dalke, A. & Schulten, K. VMD: Visual molecular dynamics. *J. Mol. Graphics* **14**, 33-38 (1996).
9. Krueger, B.P., Scholes, G.D. & Fleming, G.R. Calculation of couplings and energy-transfer pathways between the pigments of LH2 by the ab initio transition density cube method. *J. Phys. Chem. B* **102**, 5378-5386 (1998).
10. Andreussi, O., Knecht, S., Marian, C.M., Kongsted, J. & Mennucci, B. Carotenoids and Light-Harvesting: From DFT/MRCI to the Tamm-Dancoff Approximation. *J. Chem. Theory Comput.* **11**, 655-666 (2015).
11. Liu, Z.F. et al. Crystal structure of spinach major light-harvesting complex at 2.72 A resolution. *Nature* **428**, 287-292 (2004).
12. Jo, S., Kim, T., Iyer, V.G. & Im, W. Software News and Updates CHARMM-GUI : A Web-Based Graphical User Interface for CHARMM. *J. Comput. Chem.* **29**, 1859-1865 (2008).
13. Lomize, M.A., Lomize, A.L., Pogozheva, I.D. & Mosberg, H.I. OPM: Orientations of proteins in membranes database. *Bioinformatics* **22**, 623-625 (2006).
14. Ogata, K., Yuki, T., Hatakeyama, M., Uchida, W. & Nakamura, S. All-atom molecular dynamics simulation of photosystem II embedded in thylakoid membrane. *J. Am. Chem. Soc.* **135**, 15670-15673 (2013).
15. Case, D.A. et al. *{Amber 14}*, (2014).
16. Dickson, C.J. et al. Lipid14: the amber lipid force field. *J. Chem. Theory Comput.* **10**, 865-879 (2014).
17. Maier, J.A. et al. ff14SB: improving the accuracy of protein side chain and backbone parameters from ff99SB. *J. Chem. Theory Comput.* **11**, 3696-3713 (2015).
18. Prandi, I.G., Viani, L., Andreussi, O. & Mennucci, B. Combining classical molecular dynamics and quantum mechanical methods for the description of electronic excitations: The case of carotenoids. *J. Comput. Chem.* **37**, 981-991 (2016).
19. Ceccarelli, M., Procacci, P. & Marchi, M. An ab initio force field for the cofactors of bacterial photosynthesis. *J. Comput. Chem.* **24**, 129-142 (2003).
20. Zhang, L., Silva, D.A., Yan, Y. & Huang, X. Force field development for cofactors in the photosystem II. *J. Comput. Chem.* **33**, 1969-1980 (2012).
21. Wang, J., Wolf, R.M., Caldwell, J.W., Kollman, P.A. & Case, D.A. Development and testing of a general amber force field. *J. Comput. Chem.* **25**, 1157-1174 (2004).
22. Skjevik, Å.A., Madej, B.D., Walker, R.C. & Teigen, K. LIPID11: a modular framework for lipid simulations using amber. *J. Phys. Chem. B* **116**, 11124-11136 (2012).
23. Jorgensen, W.L., Chandrasekhar, J., Madura, J.D., Impey, R.W. & Klein, M.L. Comparison of simple potential functions for simulating liquid water. *J. Chem. Phys.* **79**, 926-935 (1983).
24. Joung, I.S. & Cheatham III, T.E. Molecular dynamics simulations of the dynamic and energetic properties of alkali and halide ions using water-model-specific ion parameters. *J. Phys. Chem. B* **113**, 13279-13290 (2009).
25. Schrodinger, LLC. The PyMol Molecular Graphics System, Version 1.7.4.

26. Renger, T. & Marcus, R.A. On the relation of protein dynamics and exciton relaxation in pigment-protein complexes: An estimation of the spectral density and a theory for the calculation of optical spectra. *J. Chem. Phys.* **116**, 9997-10019 (2002).
27. Polivka, T. & Sundstrom, V. Ultrafast dynamics of carotenoid excited states - From solution to natural and artificial systems. *Chem. Rev.* **104**, 2021-2071 (2004).
28. Miloslavina, Y. et al. Charge separation kinetics in intact photosystem II core particles is trap-limited. A picosecond fluorescence study. *Biochemistry* **45**, 2436-2442 (2006).
29. Belgio, E., Johnson, M.P., Juric, S. & Ruban, A.V. Higher Plant Photosystem II Light-Harvesting Antenna, Not the Reaction Center, Determines the Excited-State Lifetime-Both the Maximum and the Nonphotochemically Quenched. *Biophys. J.* **102**, 2761-2771 (2012).
